# Immunogenicity and efficacy of fowlpox-vectored and inactivated avian influenza vaccines alone or in a prime-boost schedule in chickens with maternal antibodies

**DOI:** 10.1186/s13567-014-0107-6

**Published:** 2014-10-30

**Authors:** Alexandra Richard-Mazet, Sylvain Goutebroze, François-Xavier Le Gros, David E Swayne, Michel Bublot

**Affiliations:** Merial S.A.S., R&D, 254 rue M. Mérieux, 69007 Lyon, France; Exotic and Emerging Avian Viral Diseases Research Unit, Southeast Poultry Research Laboratory, Agricultural Research Service, U.S. Department of Agriculture, 934 College Station Road, Athens, Georgia 30605 USA

## Abstract

Inactivated and fowlpox virus (FP)-vectored vaccines have been used to control H5 avian influenza (AI) in poultry. In H5 AI endemic countries, breeder flocks are vaccinated and therefore, maternally-derived antibodies (MDA) are transferred to their progeny. Results of three immunogenicity and one efficacy studies performed in birds with or without MDA indicated that the immunogenicity of an inactivated vaccine based on a H5N9 AI isolate (inH5N9) was severely impaired in chicks hatched from inH5N9-vaccinated breeders. This MDA interference was lower when breeders received only one administration of the same vaccine and could be overcome by priming the chicks at day-of-age with a live recombinant FP-vectored vaccine with H5 avian influenza gene insert (FP-AI). The interference of anti-FP MDA was of lower intensity than the interference of anti-AI MDA. The highest interference observed on the prime-boost immunogenicity was in chicks hatched from breeders vaccinated with the same prime-boost scheme. The level of protection against an antigenic variant H5N1 highly pathogenic AI isolate from Indonesia against which the FP-AI or inH5N9 alone was poorly protective could be circumvented by the prime-boost regimen in birds with either FP or AI MDA. Thus, the immunogenicity of vaccines in young chicks with MDA depends on the vaccination scheme and the type of vaccine used in their parent flocks. The heterologous prime-boost in birds with MDA may at least partially overcome MDA interference on inactivated vaccine.

## Introduction

Vaccines are useful tools to control avian influenza (AI) especially when biosecurity and stamping out strategies alone are not successfully implemented [1-3]. Inactivated vaccines are the most widely used AI vaccines although viral vectored vaccines based on fowlpox (FP) or Newcastle disease virus (NDV) are also used in some countries [4-6]. In endemic countries in which AI vaccination is routinely used in breeders, broilers hatch with maternally-derived antibodies (MDA). A few papers reported the negative effect of MDA on inactivated AI and vector vaccines immunogenicity and efficacy in chickens [7-12].

A FP-vectored AI (FP-AI) vaccine expressing the hemagglutinin (HA) gene from the A/turkey/Ireland/1378/1983 H5N8 highly pathogenic AI (HPAI) isolate has been used in broilers since 1998 in low pathogenic avian influenza (LPAI) H5N2 endemic areas of Central America. One of the main advantages of this FP-AI vaccine is that it is administered to one-day-old chicks by the subcutaneous route at the hatchery whereas inactivated AI vaccines are usually administered in the farms at a later age. The FP-AI vaccine is also a DIVA (differentiating infected from vaccinated animals) vaccine since it induces an immune response against the HA alone and some commercially available enzyme-linked immunosorbent assays (ELISA) are designed to detect the antibody response against the nucleoprotein (NP); these ELISAs can therefore detect infection in a FP-AI vaccinated flock. The FP-AI efficacy in presence of FP and/or H5 AI virus MDA has been shown in a highly pathogenic AI (HPAI) as well as low pathogenic AI (LPAI) Mexican H5N2 challenge model [7,13]. This FP-vectored vaccine was also shown to be efficacious against different HPAI H5N1 isolates in SPF chicks [14,15]. The objective of the studies presented here was to further analyse the effect of MDA on FP-AI vaccine immunogenicity and to evaluate the efficacy of a prime-boost regimen (priming with FP-AI and boosting with inactivated vaccine) in birds with FP or AI MDA.

## Material and methods

### Vaccines

Three AI vaccines were used: a classical inactivated and two recombinant FP vectored vaccines. The whole virus beta-propiolactone (BPL)-inactivated vaccine (inH5N9) contained the Eurasian isolate A/chicken/Italy/22A/98 [H5N9] (Genbank HA gene: ABR37720), propagated on embryonated SPF eggs (experimental vaccine prepared by Merial Italia S.p.A.). The inactivated antigen (H5N9-It) was formulated in a water-in-oil adjuvant similar to those used in classical avian inactivated vaccines. Two different experimental formulations of H5N9-It inactivated vaccine (inH5N9) were used. For studies 1 and 2, the vaccine was formulated at 0.5 mL (350 HA units) per dose and for studies 3 and 4, it was formulated at 0.3 mL (122 HA units) per dose. The first vectored vaccine contained an experimental FP recombinant (vFP2211) expressing a synthetic hemagglutinin (HA) gene derived from the amino acid sequence of the HA from A/chicken/Indonesia/7/2003 clade 2.1.1 H5N1 HPAI isolate (GenBank: ABO30346) that was modified at the cleavage site (RE*RRRKK*RGLF mutated into RE*T*RGLF) [7]. The other FP vectored vaccine was the TROVAC®-AIV H5 (batches TAH5RF064 (study 2), RD021 (study 3), and RD030 (study 4) produced by Merial Select, Inc., Gainesville, Georgia, USA) vaccine licensed in the USA and other countries. This vaccine contains the FP recombinant vFP89, which expresses the HA gene (Genbank: HMIVT8) of a HPAI H5N8 virus isolated in Ireland (A/turkey/Ireland/1378/83 [H5N8]). The FP vector (TROVAC® vector), insertion site and promoter were the same for the two FP vectored vaccines. It was derived from the seed of the commercial FP vaccine DIFTOSEC CT® (batch 5DCC3511 (breeders of study 1 and 2) and batch 7DCC3801-A (breeders of study 3 and 4) produced by Merial S.A.S., Lyon, France) that was also used as the FP vaccine in our studies.

### Experimental design

#### Study 1 (Immunogenicity study)

Five groups of 10 one-day-old chickens were included in this study. These birds were hatched either from unvaccinated SPF hens or from SPF hens vaccinated twice with inH5N9 (0.5 mL) at 3 and 17 weeks of age respectively. These hens were also vaccinated with FP vaccine at 9 weeks of age. Serum was collected from 15 out of 19 hens at 36 and 44 weeks of age for AI serology. The chicks hatched from eggs collected from 42 week-old vaccinated hens had either no MDA or both anti-FP and anti-H5N9 maternal antibodies (named hereafter MDA-FP + H5N9). Chicks without MDA were vaccinated with vFP2211 at 3 log_10_ TCID_50_/dose at day 0 (D0). Chicks with MDA-FP + H5N9 were vaccinated either with vFP2211 at 3 log_10_ TCID_50_/dose at D0, with vFP2211 at 5 log_10_ TCID_50_/dose at D0, with vFP2211 at 5 log_10_ TCID_50_/dose at D0 and 0.5 mL of inH5N9 at day 21 (D21) (prime-boost), or with 0.5 mL of inH5N9 at D21 only. The design of this study is shown in Table 1. Vaccination was performed subcutaneously in the nape of the neck at D0 for vFP2211 and intramuscularly in the breast at D21 for inH5N9. Blood was taken 3 weeks after the first vaccination and 3 and 9 weeks after the second vaccination to assess the seroconversion. The level of MDA was evaluated from the serum of 10 additional chicks euthanized after hatch.

**Table 1:** **Vaccination schemes of the four studies**

**Study**	**Chickens**	**1** ^**st**^ **vaccination at day 0**		**2** ^**nd**^ **vaccination at day 21 (studies 1 and 2) or at day 14 (studies 3 and 4)**		**Challenge**
		**Vaccine**	**Dose**	**Vaccine**	**Dose**	
1	SPF	vFP2211	10^3^ TCID_50_	-	-	No
	H5N9 + FP MDA	vFP2211	10^3^ TCID_50_	-	-	
		vFP2211	10^5^ TCID_50_	-	-	
		vFP2211	10^5^ TCID_50_	inH5N9	0.5 mL	
		-	-	inH5N9	0.5 mL	
2	H5N9 + FP MDA	inH5N9	0.2 ml	inH5N9	0.5 mL	No
		vFP89	10^3^ TCID_50_	inH5N9	0.5 mL	
	SPF	vFP89	10^3^ TCID_50_	inH5N9	0.5 mL	
3	SPF	vFP89	10^3^ TCID_50_	inH5N9	0.3 mL	No
		-	-	inH5N9	0.3 mL	
	H5N9 MDA	vFP89	10^3^ TCID_50_	inH5N9	0.3 mL	
		-	-	inH5N9	0.3 mL	
	FP	vFP89	10^3^ TCID_50_	inH5N9	0.3 mL	
		-	-	inH5N9	0.3 mL	
	H5(N8)* + H5N9 + FP MDA	vFP89	10^3^ TCID_50_	inH5N9	0.3 mL	
4	SPF	-	-	-	-	Yes (D28)
		vFP89	10^3^ TCID_50_	-	-	
	FP MDA	vFP89	10^3^ TCID_50_	inH5N9	0.3 mL	
		-	-	inH5N9	0.3 mL	
	H5N9 MDA	-	-	-	-	
		vFP89	10^3^ TCID_50_	-	-	
		vFP89	10^3^ TCID_50_	inH5N9	0.3 mL	
		-	-	inH5N9	0.3 mL	

Chickens were hatched from unvaccinated (SPF) or vaccinated (MDA) hens. Chicks with H5N9, FP, H5N8 and/or H5N1 MDA were the progeny of hens vaccinated with inH5N9, fowlpox (FP), vFP89 (HA gene from A/turkey/Ireland/1378/83 [H5N8]) and/or vFP2211 (HA gene from A/chicken/Indonesia/7/2003 [H5N1]), respectively. The groups of 9 to 10 one-day-old birds received either vFP2211 or vFP89 or inH5N9 at D0, followed by a boost of inH5N9 at D21 (studies 1and 2) or at D14 (studies 3 and 4). In addition, a HPAI H5N1 challenge was performed in study 4.

*H5(N8) MDA are directed against the HA only of A/turkey/Ireland/1378/83 [H5N8]. Breeders of these chicks were indeed vaccinated with a fowlpox expressing the HA of H5N8 (vFP89).

#### Study 2 (Immunogenicity study)

Three groups of 10 one-day-old chickens were included in this study. One group of SPF birds was immunized with vFP89 at 3 log_10_ TCID_50_/dose at D0 and with 0.5 mL of inH5N9 at D21 (prime-boost in SPF chickens without MDA). The birds of the two other groups were hatched from SPF hens vaccinated twice with inH5N9 (0.5 mL) at 3 and 17 weeks of age and once with FP vaccine at 9 weeks of age; serum was collected from 15 out of 19 hens at 60 and 68 weeks of age for AI serology. The chicks from these two other groups were hatched from eggs collected from 62 week-old vaccinated hens and had both anti-FP and anti-H5N9 maternal antibodies (MDA FP + H5N9). One of these 2 other groups was vaccinated with vFP89 at 3 log_10_ TCID_50_/dose at D0 and with 0.5 mL of inH5N9 at D21 (prime-boost), and the other at both D0 and D21 with inH5N9 (0.2 and 0.5 mL respectively) (see Table 1). Vaccination was performed subcutaneously in the nape of the neck at D0 and intramuscularly in the breast at D21. Blood was taken 3 weeks after the first vaccination and 1 and 3 weeks after the second vaccination to assess the seroconversion. The level of transmitted MDA was evaluated from the serum of 7 additional chicks euthanized after hatch.

#### Study 3 (Immunogenicity study)

Eight groups of 10 one-day-old chickens were included in this study. Birds were hatched from 4 different groups of hens: (1) non-vaccinated SPF, (2) SPF vaccinated 3 times with inH5N9 (0.3 mL) at 3, 6 and 16 weeks of age, (3) SPF vaccinated twice with FP vaccine at 4 and 12 weeks of age or (4) SPF vaccinated with vFP89 at 3 log_10_ TCID_50_/dose at D0, FP vaccine at 12 weeks of age and with 0.3 mL of inH5N9 at 16 weeks of age. Serum was collected from 10 out of 15 hens from group (2) and from 10 out of 20 hens of group (4) at 20 and 28 weeks of age for AI serology. The chicks were hatched from eggs collected from 24 week-old vaccinated hens and had either (1) no MDA, (2) anti-H5N9, (3) anti-FP or (4) anti-FP, anti-H5(N8) (against A/turkey/Ireland/1378/83 [H5N8] HA only) and anti-H5N9 maternal antibodies. These chicks were vaccinated with vFP89 at 3 log_10_ TCID_50_/dose at D0 and with 0.3 mL of inH5N9 at day 14 (D14) (prime-boost) or only once at D14 with 0.3 mL of inH5N9 (see Table 1). Vaccination was performed subcutaneously in the nape of the neck at D0 and intramuscularly in the breast at D14. Blood was taken 2 weeks after the first vaccination and 2 and 4 weeks after the second vaccination to assess the seroconversion. The level of MDA was evaluated from the serum of 5 additional chicks euthanized after hatch.

#### Study 4 (challenge study)

Eight groups of 9-10 one-day-old chickens were included in this study. Birds were hatched from 3 different groups of hens: (1) non-vaccinated SPF, (2) SPF vaccinated twice with FP vaccine at 4 and 12 weeks of age, (3) SPF vaccinated 3 times with inH5N9 at 3, 6 and 16 weeks of age. Serum was collected from 10 out of 15 hens from group (3) at 52 and 60 weeks of age for AI serology. The chicks were hatched from eggs collected from 58 week-old vaccinated hens and had either (1) no MDA, (2) anti-FP or (3) anti-H5N9 maternal antibodies. Chicks without MDA were vaccinated either with vFP89 at 3 log_10_ TCID_50_/dose at D0 or remained unvaccinated. Chicks with anti-FP MDA were vaccinated with vFP89 at D0 and with 0.3 mL of inH5N9 at D14 (prime-boost) or at D14 only with 0.3 mL of inH5N9. Chicks with anti-H5N9 MDA were either non-vaccinated, vaccinated with vFP89 at D0, vaccinated with 0.3 mL of inH5N9 at D14, or vaccinated with vFP89 at D0 and with 0.3 mL of inH5N9 at D14 (prime-boost) (see Table 1). Vaccination was performed subcutaneously in the nape of the neck at D0 (vFP89) and intramuscularly (inH5N9) in the breast at D14. All birds were challenged by the intranasal route at day 28 (D28) with 10^6^ EID50 per bird of the Indonesian A/chicken/West Java-Subang/29/2007 HPAI H5N1 isolate classified in 2.1.3.2 subclade [16]. The HA sequence of the A/chicken/West Java/29/002/2007 isolate available in GISAID’s EpiFlu™ Database [17] (Accession number EPI533441) was used for sequence analysis. This isolate was previously used in a published vaccination-challenge study [18] and is one of the antigenic variants that emerged in Indonesia as early as 2005 [16]. Morbidity and mortality were recorded during 2 weeks after challenge and oropharyngeal swabs were collected 2 and 4 days post-challenge to evaluate viral load by quantitative real time RT-PCR. Blood was taken 2 weeks after the first vaccination (D14) and 2 weeks after the second administration before challenge (D28). The level of MDA was evaluated from the serum of 5 additional chicks euthanized after hatch.

### Sequence and laboratory analysis

The identity and similarity between the HA1 amino acid sequence of the vaccine strains, the challenge isolate and the viruses used as antigens in the HI test (Table 2) were obtained using the Blastp software of the National Center for Biotechnology Information [19].

**Table 2:** **Amino acid sequence identity (up right) and similarity (down left) of the HA1 domain of the different H5 strains used for vaccines, challenge and/or HI test antigens**

**Identity →**	**tk/Ire/83 H5N8**	**ck/Ind/03 H5N1**	**ck/Ita/98 H5N9**	**Vtn/04 H5N1**	**tk/Tur/05 H5N1**	**ck/WJ/07 H5N1**
**Similarity ↓**						
**tk/Ire/83 H5N8**		88.0%	91.4%	88.7%	88.7%	83.4%
**ck/Ind/03 H5N1**	94.8%		89.3%	96.6%	96.0%	92.9%
**ck/Ita/98 H5N9**	96.3%	94.5%		89.6%	89.3%	85.3%
**Vtn/04 H5N1**	95.1%	97.5%	93.9%		94.8%	92.0%
**tk/Tur/05 H5N1**	94.2%	98.2%	93.9%	97.6%		90.5%
**ck/WJ/07 H5N1**	91.1%	94.8%	91.7%	94.2%	94.2%	

The HA gene of A/turkey/Ireland/1378/83 [H5N8] (tk/Ire/83 H5N8) and of A/chicken/Indonesia/7/2003 [H5N1 clade 2.1.1] (ck/Ind/03 H5N1) is inserted into the genome of fowlpox recombinant vFP89 and vFP2211, respectively. The A/chicken/Italy/22A/98 [H5N9] (ck/Ita/98 H5N9) is used in the inactivated inH5N9 vaccine. The NIBRG14 and NIBRG23 reverse genetics constructs contain the HA and NA genes from A/Vietnam/1194/2004 [H5N1 clade 1] (Vtn/04 H5N1) and from A/turkey/Turkey/1/2005 [H5N1 clade 2.2] (tk/Tur/05 H5N1), respectively. The A/chicken/West Java-Subang/29/2007 [H5N1 clade 2.1.3.2] (ck/WJ/07 H5N1) was used for the challenge in study 4.

The serological analyses were performed using the hemagglutination inhibition (HI) tests. Tests were conducted according to standard procedures, using 4 HA units/well. Different antigens were tested to check for cross-reactivity of the serums towards different recent H5N1 strains. The BPL-inactivated A/chicken/Italy/22A/98 [H5N9] (homologous to the inactivated vaccine strain) was tested on serums from all studies. The BPL-inactivated NIBRG14 H5N1 antigen was used on serums from studies 1 and 2; the NIBRG14 is a 6/8 PR8-based reverse genetics mutant containing the HA (Genbank: ACR48874) and NA of the A/Vietnam/1194/2004 clade 1 H5N1 isolate. The BPL-inactivated NIBRG23 H5N1 antigen was used on serums from study 3; the NIBRG23 is a 6/8 PR8-based reverse genetics mutant containing the HA (Genbank: ABQ58921) and NA of the A/turkey/Turkey/1/2005 clade 2.2 H5N1 HPAI isolate. The H5N8 A/turkey/Ireland/1378/83 antigen, homologous to the HA used in the vFP89 vaccine was also used in studies 2, 3 and 4 and the BPL-inactivated A/chicken/West Java-Subang/29/2007 was used in study 4. The serums of breeders were also tested with the H5N9 antigen as well as with the NIBRG14 (studies 1 and 2) or NIBRG23 (studies 3 and 4) H5N1 antigen. The HI tests on serums from breeder hens were performed at Merial; those from their progeny of studies 1 to 3 and from study 4 were performed at Merial and SEPRL, respectively. The first tested serum dilution was 1 log2 (studies 1 to 3) and 3 log2 (study 4).

The level of viral shedding after challenge was evaluated in study 4 by M-based real time reverse transcriptase (RT) PCR on RNA extracted from swabs as previously described [20].

Statistical analyses were performed using the STATGRAPHICS and SAS/PC softwares. Analysis were carried out by applying the one-way analysis of variance (1-ANOVA) or two-way analysis of variance (2-ANOVA) with factors “origin of the birds” and “vaccination scheme” and pairwise multiple comparison procedure by the Student’s *t*-test. Statistical difference was based on two-tailed tests of the null hypothesis resulting in p-value of 0.05 or less. A value of 0 (studies 1-3) and 2 log2 (study 4) were set for serums that tested negative for statistical analysis.

## Results

### Antibody titers in the breeders and progeny

Mean H5N1 and H5N9 HI titers in the breeder hens before and after egg harvest and in their one-day-old progeny are presented at Table 3 for the 4 studies. The mean HI titers in the progeny were lower (by 1 to 4 log2) than in their parent hens.

**Table 3:** **H5N1 and H5N9 HI titers in breeders and their progeny**

**Study**	**Type of breeders (age at egg harvest)**	HI titers* **in breeders**			**HI titers in progeny**	
		**Age at blood sampling**	**H5N1**	**H5N9**	**H5N1**	**H5N9**
1	H5N9 + FP (42 weeks)	36 weeks	4.0 ± 1.8	7.5 ± 2.2	2.0 ± 2.2	3.5 ± 3.0
		44 weeks	2.9 ± 2.1	7.5 ± 2.5		
2	H5N9 + FP (62 weeks)	60 weeks	3.5 ± 1.9	6.5 ± 2.7	1.0 ± 1.7	3.1 ± 2.7
		68 weeks	3.0 ± 1.9	6.6 ± 2.1		
3	H5N9 (24-25 weeks)	20 weeks	4.9 ± 2.0	7.1 ± 1.7	3.0 ± 1.9	4.3 ± 2.0
		28 weeks	4.3 ± 2.5	5.9 ± 2.0		
	H5(N8) + H5N9 + FP (24-25 weeks)	20 weeks	ND**	7.0 ± 1.6	1.8 ± 1.1	3.0 ± 1.9
		28 weeks	ND	5.3 ± 1.9		
4	H5N9 (58 weeks)	52 weeks	3.9 ± 2.7	5.0 ± 2.1	ND	3.4 ± 0.9
		60 weeks	4.6 ± 2.2	5.8 ± 2.3		

Breeders hens were vaccinated with inH5N9 and fowlpox (H5N9 + FP), inH5N9 only (H5N9) or with vFP89, inH5N9 and fowlpox (H5(N8) + H5N9 + FP). Their serums were taken at two time points flanking the time of harvest of their eggs. Serums of their progeny were sampled at 1 day-of-age. NIBRG14 and NIRG23 H5N1 antigens were used in studies 1 & 2 and studies 3 & 4, respectively.

*mean HI titer ± standard deviation in log2.

**ND means not done.

### Study 1

**Figure 1:**
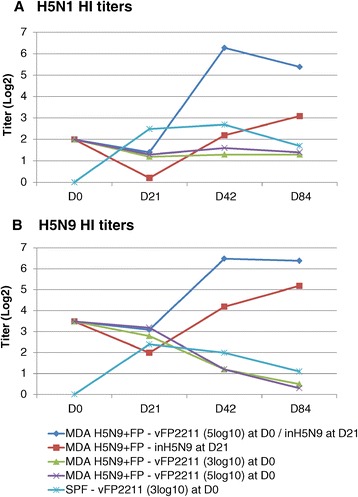
**Study 1 Kinetics of HI antibody titers induced by different vaccines**. Mean HI titers (log2) using **(A)** H5N1 clade 1 (NIBRG14) and **(B)** H5N9 antigens in the serum of five groups of vaccinated chickens. One group of SPF chickens was vaccinated with 3 log10 of vFP2211. The four other groups had H5N9 and FP MDA and were vaccinated either with 3 or 5 log10 of vFP2211 at D0, with inH5N9 at D21, or with 5 log10 of vFP2211 at D0 followed by inH5N9 at D21 (prime-boost). HI titers were evaluated at D0, D21 (3 weeks after the vaccination with vFP2211), D42 and D84 (3 and 9 weeks, respectively, after the 2^nd^ vaccination with inH5N9 in the prime-boost group).

Assessment of seroconversion was performed at the time of first (D0) or second (D21) vaccination and after the second vaccination (day 42 (D42) and 84 (D84)). Mean HI titers using two different antigens (H5N1 clade 1 and H5N9) are presented in Figure 1 and the HA1 amino acid homologies between the vaccine and HI antigens are shown in Table 2. The vFP2211 at 3 log10 TCID50/dose induced a low antibody response (2-3 log2) with both antigens that peaked between D21 and D42 in SPF chicks without MDA. In birds with FP/H5N9 MDA, the induced antibody response was decreased by 1-2 log2 suggesting an interference of MDA on vFP2211 immunogenicity. This difference was statistically significant (*p* = 0.046) with the H5N1 antigen at D21. Increasing the vFP2211 dose to 5 log10 did not increase the HI titers in presence of MDA. One administration of the inH5N9 vaccine in birds with MDA induced an antibody response of 5 and 3 log2 against homologous H5N9 and heterologous H5N1 antigens, respectively. When birds were primed with vFP2211 (dose of 5 log10), the antibody titers after the inH5N9 boost were significantly higher than after the inH5N9 alone. Interestingly, titers against both H5N1 and H5N9 antigens were similar. Overall, results of study 1 indicated a slight interference of FP and/or H5N9 MDA on vFP2211 immunogenicity. However, vFP2211 priming before inH5N9 administration significantly increased the HI titers after the boost indicating an efficient vFP2211 priming despite the presence of MDA.

### Study 2

**Figure 2:**
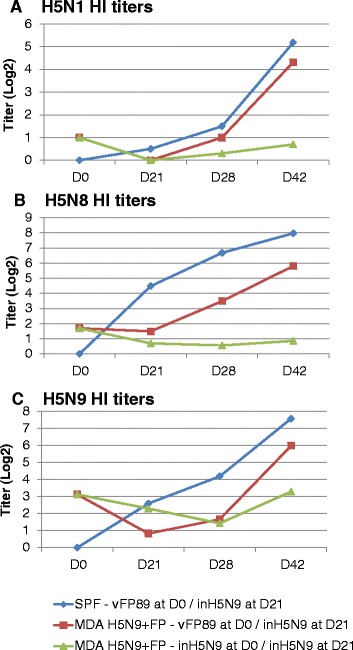
**Study 2 Kinetics of HI antibody titers induced by different vaccines.** Mean HI titers (log2) using **(A)** H5N1 clade 1 (NIBRG14), **(B)** H5N8 and **(C)** H5N9 antigens on D0, D21 (3 weeks after the 1^st^ vaccination), D28 and D42 (1 and 3 weeks after the 2^nd^ vaccination, respectively). Two groups of chicks with H5N9 and FP MDA were vaccinated either with vFP89 at D0 followed by inH5N9 at D21 (prime-boost) or inH5N9 at both D0 and D21. The third group was SPF chicks without MDA vaccinated with the prime boost.

Assessment of seroconversion induced by a homologous (inH5N9 + inH5N9) or a heterologous (vFP89 + inH5N9) prime-boost scheme was performed at the time of first (D0) or second (D21) vaccination or after the second vaccination (D28 and D42) in SPF chickens or chickens with FP and H5N9 MDAs. Results of the HI test using three different antigens (H5N1 clade 1, H5N8 and H5N9) are presented in Figure 2 and the HA1 amino acid homologies between the vaccine and HI antigens are shown in Table 2.

After the first inH5N9 vaccine administration at D0, no clear seroconversion was observed in birds with MDA at D21. In SPF chicks, the vFP89 induced at D21 a mean homologous H5N8 HI titer of 4.5 log2. However, no H5N8 seroconversion was observed with this vaccine in birds with MDA indicating that FP and/or AI MDA had negative impact on vFP89 AI immunogenicity.

In presence of anti-FP and anti-H5N9 maternal antibodies, the group vaccinated at both D0 and D21 with inH5N9 had surprisingly no seroconversion against the H5N1 and H5N8 antigens and a very low seroconversion against the H5N9 homologous antigen after the 2^nd^ administration suggesting an interference of the MDA on the inH5N9 immunogenicity. In contrast, the group receiving the vFP89 priming at D0 followed by inH5N9 at D21 clearly showed a seroconversion in presence of MDA for the 3 antigens that was significantly higher at D28 for the H5N8 antigen and at D42 for all 3 antigens than the groups receiving 2 times inH5N9. However, the same prime-boost scheme in SPF chicks induced higher HI titers than in birds with H5N9 + FP MDA. The HI titers in SPF without MDA were significantly higher with H5N8 and H5N9 antigens at both D28 and D42 (Kruskal-Wallis test H5N8 antigen: *p* < 0.01 and *p* = 0.04, respectively; H5N9 antigen: *p* = 0.02 and *p* = 0.04, respectively) but not with the heterologous H5N1 antigen (Kruskal-Wallis test: *p* = 0.71 and 0.69, respectively). Note that the identity and similarity between the HA1 amino acid sequence of the vaccines and the H5N1 clade 1 (NIBRG14) HI antigen was lower or equal to 90% and 95%, respectively (Table 2).

### Study 3

Chickens with or without MDA (SPF) were used in this 3^rd^ study. Chicks with MDA had either (1) H5N9 MDA, (2) FP MDA or (3) H5(N8) + H5N9 + FP MDA (see Materials and methods). They were vaccinated with vFP89 at D0 and inH5N9 at D14 or with inH5N9 only (see Table 1). Assessment of seroconversion in the vaccinated birds was performed at D0, D14, D28 and D42. HI test was performed with three different antigens (H5N1 clade 2.2, H5N8 and H5N9). The HA1 amino acid homologies between the vaccines and the H5N1 clade 2.2 (NIBRG23) HI antigen were similar than between the vaccines and the H5N1 clade 1 (NIBRG14) antigen used in studies 1 and 2 (Table 2).

**Figure 3:**
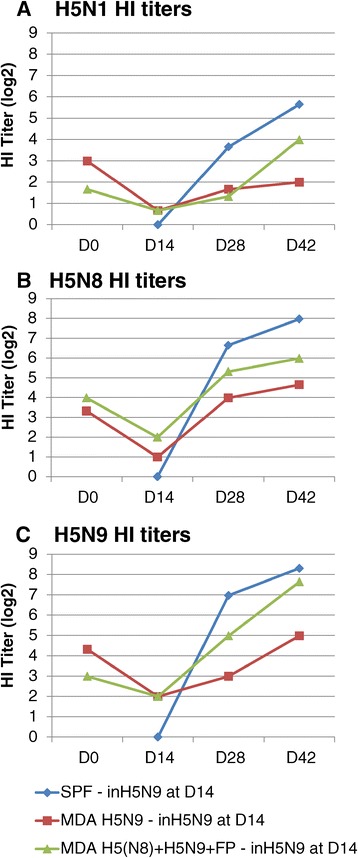
**Study 3 Kinetics of HI antibody titers induced by the inH5N9 vaccine.** Mean HI titers (log2) using **(A)** H5N1 (NIBRG23), **(B)** H5N8 and **(C)** H5N9 antigens on D0, D14, D28 and D42 in groups vaccinated with inH5N9 at D14. The birds of these three groups were SPF (no MDA) or with H5N9 MDA or with H5(N8) + H5N9 + FP MDA.

**Figure 4:**
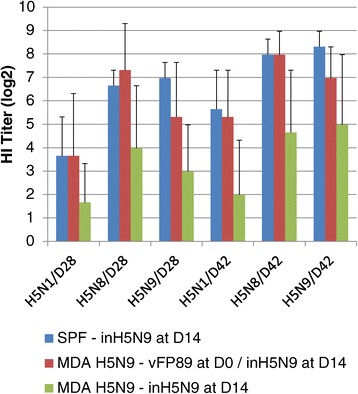
**Study 3 Overcoming MDA-interference on inH5N9 vaccine by vFP89 priming.** Mean HI titers (log2) using H5N1 (NIBRG23), H5N8 and H5N9 antigens on D28 and D42 (2 and 4 weeks after the inH5N9 vaccination). Birds were either SPF without MDA or had H5N9 MDA and were vaccinated with vFP89 at D0 and inH5N9 at D14 (birds with MDA) or inH5N9 at D14 only (birds with or without MDA).

The HI titre kinetics in SPF, H5N9 MDA or H5(N8) + H5N9 + FP MDA birds vaccinated only once with inH5N9 at D14 is shown in Figure 3. A clear interference of AI MDA was observed on the immunogenicity of inH5N9 vaccine and this interference was higher in H5N9 MDA chicks than in H5(N8) + H5N9 + FP MDA chicks, the mean HI titers at D42 being 3-4 log2 below those obtained in SPF birds. Indeed, HI titres in H5N9 MDA chicks at D42 were significantly lower (t-test: *p* = 0.022 and *p* < 0.001 for H5N1 and H5N9 antigens, respectively) than those obtained in H5(N8) + H5N9 + FP MDA chicks; at D28, a lower HI titer in H5N9 MDA versus H5(N8) + H5N9 + FP MDA birds was also observed for both H5N8 and H5N9 antigens but the difference was significant (*p* = 0.003) with the H5N9 antigen only. These results suggest that the level of MDA interference on inH5N9 immunogenicity depends on the number of inH5N9 administrations given to the breeders. In chicks with H5N9 MDA, the vFP89 priming at D0 could clearly overcome the interference of H5N9 MDA on inH5N9 vaccine D14 immunogenicity, since on both D28 and D42 and for the 3 antigens, the HI titres were significantly higher than those induced after one inH5N9 administration to H5N9 MDA chicks (t-test: *p* = 0.02, *p* = 0.002 and *p* = 0.003 at D28, and *p* = 0.01, *p* = 0.0001 and *p* = 0.017 at D42 for H5N1, H5N8 and H5N9 antigens, respectively) and not different (*p* > 0.05) from those induced after one inH5N9 administration to SPF chicks (see Figure 4).

**Figure 5:**
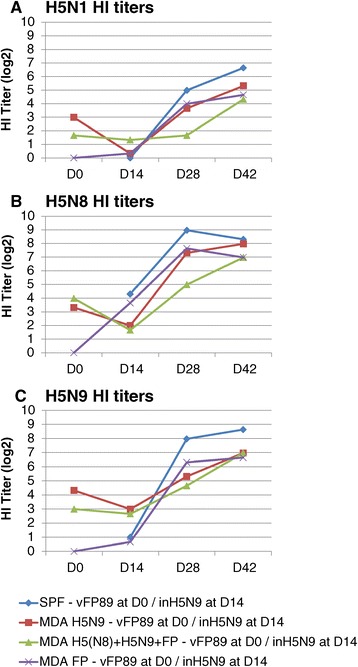
**Study 3 Kinetics of HI antibody titers induced by the prime-boost.** Mean HI titers (log2) using H5N1 **(A)**, H5N8 **(B)** and H5N9 **(C)** antigens on D0, D14, D28 and D42 in groups vaccinated with the prime-boost (vFP89 at D0 and inH5N9 at D14). The birds of these four groups were SPF or with H5N9 MDA or with FP MDA or with H5(N8) + H5N9 + FP MDA.

HI titer kinetics of birds with or without MDA vaccinated with the prime-boost scheme are shown in Figure 5. The highest antibody response against the 3 antigens was observed in SPF chicks without MDA. A clear MDA interference was observed mainly on D28 in chicks with H5(N8) + H5N9 + FP MDA, for which the titres were around 2 log2 lower than those of the groups with other types of MDA (see Figure 5). This interference was less pronounced on D42. The statistical analysis confirmed this difference on D28, HI titres from chicks with H5(N8) + H5N9 + FP MDA being significantly lower than those in SPF chicks for the three antigens (t-test: *p* = 0.006, *p* = 0.002 and *p* = 0.004 for H5N1, H5N8 and H5N9 antigens, respectively), lower than those in chicks with H5N9 MDA with the H5N8 antigen only (t-test: *p* = 0.028) and lower than those in chicks with FP MDA with the H5N1 and H5N8 antigens (t-test: *p* = 0.024 and *p* = 0.008, respectively).

In chicks with FP MDA, vFP89-induced H5N8 HI titres at D14 were only slightly (non-significant difference of about 0.6 log2) lower than those obtained in SPF chicks (see Figure 5B). In contrast, these H5N8 HI titres induced by vFP89 at D14 were significantly lower (about 3 log2) in chicks with H5N9 or H5(N8) + H5N9 + FP MDA compared to SPF and FP-MDA birds. These data indicate that the anti-HA insert AI MDA had more inhibitory effect on vFP89-induced H5N8 HI titers than the anti-vector FP MDA. After the inH5N9 boost, HI titres were slightly lower than in SPF (1-2 log2 difference significantly different at D42 and for H5N9 antigen only) suggesting a slight interference of the FP MDA on the vFP89 priming efficiency. In chicks with H5N9 MDA, the HI titers induced by the prime-boost regimen were lower than in SPF but the difference was statistically significant (*p* = 0.021) at D28 and for the H5N9 antigen only.

### Study 4

Eight groups of chicks with FP, with H5N9 or without (SPF) MDA were used in this 4^th^ study (see Materials and methods and the design in Table 1). Results of serology with H5N8 and H5N9 antigens, clinical protection and shedding after challenge are summarized in Table 4. Results of serology with the A/chicken/West Java-Subang/29/2007 [H5N1 clade 2.1.3.2] challenge strain antigen are not shown since all serums were negative (titer < 3 log2) before challenge (D28). The HA1 amino acid homology of the vaccines with theH5N1 clade 2.1.3.2 challenge strain was indeed lower than with the H5N1 clade 1 or 2.2 antigens (Table 2). Furthermore, the HA1 amino acid sequence of this challenge strain had mutations at critical positions adjacent to the receptor binding site: in particular, it had the S133A and A185E mutations responsible for the antigenic drift of the A/chicken/West Java/30/2007 isolate, and one (I151T) of the 4 significant changes responsible for the antigenic drift of the A/chicken/West Java/119/2010 isolate [16]. The number of positive birds (HI titer ≥ 3log2) and the mean HI titer ± standard deviation in 5 birds with H5N9 MDA sacrificed at 1 day of age were 5/5 at 4.2 ± 1.1 log2 and 4/5 at 3.4 ± 0.9 log2 with the H5N8 and the H5N9 antigens, respectively. After challenge with the Indonesian H5N1 antigenic variant HPAI, all 10 SPF and all 9 MDA H5N9 unvaccinated control birds died 2 days post-challenge (dpc) (mean death time (MDT) of 2.0) confirming the severity of the challenge and the absence of H5N9 MDA protection at 28 days of age. At 2 dpc, high levels of oropharyngeal viral excretion were detected for all unvaccinated control birds (7.8 and 7.3 log10 EID50-equivalents in chicks without or with H5N9 MDA, respectively).

**Table 4:** **Results of the vaccination-challenge study (Study 4)**

**Group**	**MDA**	**Vaccine**		**H5N8 HI titers** ^**a**^		**H5N9 HI titers**	**Protection** ^**b**^	**Oropharyngeal shedding** ^**c**^	
		**D0**	**D14**	**D14**	**D28**	**D28**	**(MDT)**	**2 Dpc**	**4 Dpc**
1	-	-	-	ND^d^	0/10 (2.0 ± 0.0)	0/10 (2.0 ± 0.0)	0/10 (2.0)	10/10 (7.8 ± 0.3)	ND
2	-	vFP89	-	10/10 (3.7 ± 0.8)	10/10 (5.5 ± 1.2)	8/10 (2.9 ± 0.6)	1/10 (3.4)	10/10 (6.3 ± 1.3)	5/5 (6.6 ± 0.7)
3	FP	vFP89	inH5N9	2/9 (2.3 ± 0.7)	9/9 (8.1 ± 1.6)	9/9 (7.6 ± 1.8)	9/9	6/9 (4.6 ± 0.7)	4/9 (4.0 ± 0.5)
4	FP	-	inH5N9	ND	9/9 (5.1 ± 1.6)	9/9 (5.8 ± 0.7)	5/9 (7.3)	7/9 (4.9 ± 0.9)	4/9 (4.6 ± 1.0)
5	H5N9	-	-	ND	0/9 (2.0 ± 0.0)	0/9 (2.0 ± 0.0)	0/9 (2.0)	9/9 (7.3 ± 0.1)	ND
6	H5N9	vFP89	-	ND	5/9 (2.9 ± 0.9)	0/9 (2.0 ± 0.0)	0/9 (2.7)	9/9 (7.1 ± 0.5)	1/1 (7.7)
7	H5N9	vFP89	inH5N9	0/9 (2.0 ± 0.0)	9/9 (5.8 ± 1.0)	9/9 (4.3 ± 1.1)	7/9 (7.0)	6/9 (4.5 ± 0.6)	7/9 (4.6 ± 1.3)
8	H5N9	-	inH5N9	0/9 (2.0 ± 0.0)	4/9 (3.2 ± 1.6)	6/9 (3.7 ± 1.7)	4/9 (5.0)	8/9 (5.0 ± 1.0)	7/8 (5.3 ± 1.1)

Results of serology, protection and oropharyngeal viral shedding in chicks with or without FP or H5N9 MDA vaccinated or not with vFP89 and/or inH5N9 and challenged at D28 with an HPAI H5N1 Indonesian isolate (A/chicken/West Java-Subang/29/2007).

^a^Number of positive birds (HI titer ≥ 3 log2)/total; the mean HI titer ± standard deviation is given between brackets.

^b^Number of birds protected against mortality/total; the mean death time (MDT) is given between brackets.

^c^Number of positive birds (≥3.8 log10 EID50-equivalent)/total; the mean viral RNA load ± standard deviation is given in log10 EID50-equivalent between brackets; Dpc, day post-challenge.

^d^ND means not done.

The vFP89 alone induced homologous H5N8 HI titers at D14 (3.7 log2 mean HI titer) and D28 (5.5log2) in all 10 SPF birds of group 2. In birds with H5N9 MDA, H5N8 seroconversion after vFP89 vaccination was observed in none (0/9) of the birds at D14 (group 7) and in only 5/9 birds (2.9 log2) at D28 (group 6) indicating an interference of H5N9 MDA on vFP89 immunogenicity. In birds with FP MDA, 2/9 birds had detectable H5N8 antibodies (2.3 log2) 14 days after vFP89 vaccination. This decrease of vFP89 immunogenicity in FP MDA birds compared to SPF birds was higher than in study 3 (1.4 log2 and 0.6 log2 decrease in study 4 and 3, respectively). Only one out of ten SPF birds without MDA was clinically protected after the vFP89 vaccine alone (MDT 3.4), whereas none of the 9 vFP89-vaccinated birds with H5N9 MDA survived after challenge (MDT 2.7); this difference was not significant. Viral shedding was detected in all vFP89-vaccinated challenged birds at similar levels as non-vaccinated control (7.1 and 7.7 log10 at 2 and 4 dpc, respectively) in birds with H5N9 MDA (group 6) and at slightly lower levels (6.3 and 6.6 log10 at 2 and 4 dpc, respectively) in SPF birds (group 2). These results indicated a low level of protection induced by vFP89 alone against this variant Indonesian HPAI H5N1 isolate which may further be decreased by the presence of AI MDA.

The inH5N9 alone induced higher homologous H5N9 HI titers and in a higher proportion of birds (mean titer of 5.8 log2; 9 positive/9 birds) at D28 in FP (group 4) compared to H5N9 MDA (group 8) chickens (mean titer of 3.7 log2; 6 positive/9 birds) confirming the negative impact of H5N9 MDA on the inH5N9 immunogenicity. After challenge, 5/9 or 4/9 chickens with FP (group 4) or H5N9 MDA (group 8), respectively, were clinically protected. The mean death time was higher in group 4 (7.3) than in group 8 (5.0). There was no relation between D28 HI titers and clinical protection for group 4 (inH5N9 at D14 in FP MDA) since dead birds pre-challenge H5N8 and H5N9 HI titers (4-6 and 6 log2, respectively) were similar than those in protected birds (4-9 and 5-7 log2, respectively). Interestingly, such relation existed for group 8 (inH5N9 at D14 in H5N9 MDA): all 5 dead birds were negative (<3 log2) with H5N8 antigen, and negative (3 birds) or at minimum titer (3 log2 in 2 birds) with homologous H5N9 antigen whereas all protected birds had detectable D28 H5N8 and H5N9 HI titers ranging from 4 to 6 log2. The viral shedding at 2 dpc of groups 4 and 8 was comparable (about 5 log10 in all but 1 or 2 birds) and 2.9 or 2.3 log10 lower than the unvaccinated controls in FP or H5N9 MDA chickens, respectively. The shedding at 4 dpc was slightly lower in group 4 (mean titer of 4.6 log10 detectable in 4/9 birds) than in group 8 (mean titer of 5.3 log10 detectable in 7/8 birds). No relation was found between D28 HI titers and the level of shedding. These results indicate that this experimental inH5N9 vaccine was only partially protective against this Indonesian HPAI H5N1 challenge and that H5N9 MDA decreased its immunogenicity.

The groups that received the heterologous prime-boost regimen in presence of FP (group 3) or H5N9 (group 7) MDA induced higher HI titers (7.6 vs 5.8 in FP MDA birds and 4.3 vs 3.7 log2 in H5N9 MDA birds) and/or in a higher number of birds (9/9 vs 6/9 in H5N9 MDA birds) than birds that received the inH5N9 only (groups 4 and 8). These birds of group 3 and 7 were also better clinically protected (9/9 and 7/9 (MDT 7.0), respectively) than those of groups 4 and 8 (5/9 (MDT 7.3) and 4/9 (MDT 5.0), respectively). The levels of viral shedding of groups 3 and 7 were comparable to those of groups 4 and 8, respectively. There was no relation between HI titers at D42 and clinical protection or shedding level in birds from groups 3 and 7. The prime-boost regimen improved the immunogenicity and protection levels compared to one inH5N9 administration for all tested condition (anti-vector FP MDA and anti-H5N9 MDA).

## Discussion

Most published studies on the efficacy of avian influenza vaccines in chickens are performed in SPF chickens without MDA. In endemic countries in which vaccination is practiced, breeder flocks are heavily vaccinated due to their very high economic value in both egg and poultry meat productions. Consequently, breeder hens transfer high MDA levels to their progeny which may protect the hatched chicks but may also interfere with vaccine efficacy. Therefore, the level of immunogenicity and protection induced by vaccination in the presence of MDA is important to evaluate. The study of MDA interference is complex since many factors can influence the data including (1) the vaccination protocol and the types of vaccines used in the breeders, (2) the time between immunization of breeder hens and collection of their eggs, (3) the type of birds and in particular their growth rate which influences the kinetics of MDA decrease after hatching, (4) the heterogeneity of MDA levels in the progeny, (5) the age of vaccination and the type of vaccine used in the progeny, and (6) the timing of challenge with a possible protective effect of MDA. Using vector vaccines, both MDA against the vector and against the targeted disease agent need to be evaluated since both may potentially interfere with immunogenicity. Our objective was to evaluate the MDA interference on FP-vectored and inactivated H5 vaccines’ immunogenicity and protection. Different vaccination schemes were tested and compared in SPF chicks and chicks with MDA.

Mean HI titers in 1-day-old chicks were lower (by 1 to 4 log2) than those in their parent hens. A similar difference (2.1-2.5 log2) in homologous H5N2 HI titers between the serum of vaccinated broiler breeder hens and their progeny was previously reported [21,22]. The difference is probably due to a low level (about 10%) of embryo IgY absorption from the egg yolk [23]. The low titer in 1-day-old chick may also be due to the incomplete resorption of the yolk sac in the newly hatched chicks. Antibody continues to be absorbed from the yolk sac after hatching [24] and total serum IgY levels were shown to increase to their maximum value at about 2 days post-hatch [25]. We have observed similar and even higher mean HI titers (up to 1.7 log2 higher) between day of hatch and one week-of-age in SPF white Leghorn type of birds hatched from vaccinated hens (Bublot et al., unpublished results). The small number of birds used to evaluate the mean HI titers in hens and one-day-old chicks may also explain the variable difference between these hen and progeny titers in different studies. Measuring the antibody titer after a couple days of live and in a higher number of animals could give a more accurate level of MDA.

Results of study 1 indicated that FP and/or H5N9 MDA can slightly interfere with vFP2211 immunogenicity. In addition, increasing the dose of vFP2211 by 2 log10 did not induce an increase in HI titers. The immunogenicity of the inH5N9 alone in birds with FP and H5N9 MDA was lower than expected in both studies 1 and 2 but there was no control group of SPF birds receiving the inH5N9 alone to show that this lower immunogenicity was due to MDA interference. However, vFP2211 priming before inH5N9 administration significantly increased the HI titers after the boost in study 1 indicating an efficient vFP2211 priming despite the presence of FP and H5N9 MDA. Interestingly, the HI titers against both H5N9 and H5N1 antigens were similar after the prime-boost. In study 2, the heterologous prime-boost vaccination regimen using the vFP89 + inH5N9 combination clearly induced a higher HI antibody response than the homologous inH5N9 + inH5N9 prime-boost in birds with MDA confirming the superior immunogenicity of the heterologous prime-boost compared to two administrations of inH5N9 vaccine in birds with MDA. However, the immune response induced by the heterologous vFP89 + inH5N9 prime-boost in birds with FP and H5N9 MDA was lower than in SPF birds indicating that MDA interfered on this vaccination scheme.

In the first two studies, chicks with both AI and FP MDA were used and therefore it was not possible to determine if the interference observed was due to the anti-vector and/or to the anti-AI maternal immunity. In addition, the MDA interference on the inH5N9 immunogenicity was not assessed. Studies 3 and 4 were therefore designed to answer to these questions in terms of immunogenicity (both studies 3 and 4) and efficacy (study 4). In study 3, the highest MDA interference (3-4 log2 lower than in SPF at D28 and D42) was observed on the immunogenicity of the inH5N9 vaccine given at 14 day-of age to birds hatched from breeders vaccinated three times with the same inH5N9 vaccine. Interestingly, in birds hatched from breeders vaccinated with a prime-boost regimen including only one administration of inH5N9, the interference was lower (see Figure 3), indicating that the number of vaccination applied to the breeders may influence the immunogenicity of the same vaccine given to the progeny. These results supported the hypothesis that the poor immunogenicity of the inH5N9 vaccine observed in studies 1 and 2 was due to MDA interference. A similar but less important (2 log2) interference of inH5N9 MDA on the inH5N9 immunogenicity was observed in study 4 (groups 4 and 8 in Table 4); this lower interference was probably due to the lower H5N9 MDA titers in chicks from study 4 (Table 3) because the timing of harvest of eggs from breeders was much later in study 4 (58-week-old breeders) than in study 3 (24-week-old breeders). The H5N1 HPAI protection induced by one-shot (D14) of inH5N9 vaccine in birds with or without H5N9 MDA was not significantly different in the tested conditions of study 4. However, birds with H5N9 MDA (group 8) tended to be less protected than those without H5N9 MDA (group 4): (1) one additional bird died (5/9 vs 4/9), (2) mean time to death was 2.3 days shorter (5.0 vs 7.3) and (3) the number of birds shedding the virus at 2 and 4 dpc was higher (total of 15/17 vs 11/18) (Table 4). Interestingly, there was a clear relation between H5N8 and H5N9 HI titers at D28 and clinical protection in birds with H5N9 MDA (all birds with a H5N8 and H5N9 titer ≥ 4 log2 were protected and those with a titer ≤ 3 log2 died) but not in birds with FP MDA (dead birds had similar H5N8 and H5N9 titers (4-6 log2) than protected ones (4-9 log2)). Prediction of protection based on H5N9 and H5N8 HI titers before this Indonesian antigenic variant challenge was therefore valid in birds with but not in birds without H5N9 MDA. At similar inH5N9-induced H5N9 and H5N8 HI titers (4-6 log2) before challenge, all 4 birds hatched with H5N9 MDA were protected whereas only 4/8 birds with no AI MDA resisted the challenge. Additional studies need to be performed to confirm these results and to identify the immune response differences that lead to the same HI titers but a different level of protection in birds with or without AI MDA.

Passive antibody interference on AI inactivated vaccines immunogenicity and efficacy has been reported by different teams. The MDA impact on inactivated vaccine immunogenicity was a drop in HI titers ranging from 2 log2 [11] to up to 8 log2 [10]. Protection levels were however only slightly reduced in birds with MDA after H5N1 HPAI challenge [11]. Abdelwhab et al. [22] showed that the MDA interference on immunogenicity was higher when the same vaccine was used in the breeders and in the progeny, and when the breeder vaccine antigen was used in the HI test. Our data showing that the MDA interference level was dependent on the breeder vaccination program are in line with these findings. The MDA interference on inactivated vaccine immunogenicity was also strongly suspected in Indonesian broilers when the same vaccine was used in breeders and their progeny [26]. Kim et al. [9] and Forrest et al. [27] simulated the presence of MDA by passive transfer of hyperimmune serum in young chicks and showed that such transfer also interfered on the immunogenicity and efficacy of commercial inactivated vaccines. Altogether, these data confirm that MDA transferred from the vaccinated breeders to their progeny interfere with the immunogenicity and efficacy of inactivated vaccine given at least in the first 2 weeks of age, especially when multiple administrations of the vaccine are used in breeders and the same vaccine is used in the progeny. The interference is detected even with low MDA titers and is lower when heterologous inactivated vaccines are used in the progeny. It is therefore important to take into account this interference when designing the vaccination program of breeders and their progeny.

The anti-FP and anti-AI MDA interference on vFP89 immunogenicity could be evaluated only before the boost (D14) in studies 3 and 4. Anti-FP MDA had only a slight negative effect on vFP89-induced H5N8 HI titers in study 3, but in study 4, the negative effect of anti-FP MDA was higher. After the inH5N9 boost in vFP89-primed birds with FP MDA in study 3, the HI titers did not reach the levels obtained after prime-boost immunization of SPF birds (Figure 5). These results suggest that the vFP89 priming efficiency may have been partially inhibited by the FP MDA. The priming effect of vFP89 on inH5N9-induced immunogenicity and protection (study 4; see Table 4) in birds with FP MDA was obvious since the mean HI titer at D28 and the number of protected birds were higher with vFP89 priming (group 3) than in the group without vFP89 priming (group 4). In previous studies performed with vFP89 [7] and with another FP recombinant expressing Newcastle disease virus (NDV) protective genes [28], no interference of FP MDA could be observed on AI and ND protection. Altogether, these data indicate that there may be a slight negative interference of anti-FP MDA on the immunogenicity of FP vectors but the anti-vector MDA effect on protection seems minor. This low interference of passive anti-FP immunity contrasts with the strong one observed in birds with active FP immunity. Both Swayne et al. [29] and Iritani et al. [30] have shown in an AI and a NDV model, respectively, that FP vaccination performed before administration of the FP recombinants severely decreased the induced protection. The low interference of passive FP immunity could be due to the poor level of FP-induced neutralizing antibodies [31], and the high interference of active FP immunity to the high FP-induced cellular immunity [32].

The interference of anti-AI MDA on vFP89-immunogenicity in both study 3 and study 4 was more obvious than that of anti-FP MDA. A lower number of seropositive birds and a lower mean HI titer (by approximately 2.5 log2) were detected compared to SPF. These data suggest that interference of MDA directed against both FP and AI observed in studies 1 and 2 on vFP2211 and vFP89 immunogenicity may rather be due to anti-AI MDA than to anti-FP MDA, without excluding the impact of anti-FP MDA. No conclusion can be drawn on the effect of H5N9 MDA on vFP89 efficacy since in the conditions tested in study 4, the vFP89 alone protected only 1/10 SPF birds and 0/9 birds with H5N9 MDA. The poor performance of vFP89 in SPF birds against this Indonesian H5N1 HPAI was surprising since several efficacy studies ([14,15,31], M. Bublot, D.E. Swayne and T. van den Berg, unpublished data) performed previously in SPF chickens with other H5N1 HPAI (clade 0, 1, 2.2 and 2.5) showed efficacy levels ranging from 75-100%. The low vFP89 efficacy level in SPF observed here is likely due to the antigenic drift of this Indonesian isolate that contains critical mutations adjacent to the receptor binding site of its HA1 that were shown to be responsible for antigenic drift in Indonesia [16]. A similar poor level of protection induced by inactivated vaccines containing seed strains A/turkey/Wisconsin/1968 (H5N9), A/chicken/Mexico/28159-232/1994 (H5N2), A/turkey/England/N28/1973 (H5N2), A/chicken/Legok/2003 (H5N1), reverse genetic A/goose/Guangdong/1/96 (H5N1), or reverse genetic A/chicken/Vietnam/C57/2004 (H5N3) against another Indonesian antigenic variant (A/chicken/West Java/PWT-WIJ/2006 (H5N1 clade 2.1.3)) has been observed (D.E. Swayne et al., manuscript in review). In previous studies performed with vFP89 in broilers with both AI and FP MDA, there was no significant interference of the MDA on the protection induced against a HPAI H5N2 Mexican challenge model [7]. However, Faulkner et al. [12] showed recently the interference of passively transferred antibodies on the immunogenicity and H5N1 HPAI protection induced by fowlpox- or NDV-vectored AI vaccines indicating that the level of interference likely depends on the tested conditions. A slight interference of MDA directed against the foreign gene product expressed by the vector vaccine has also been reported for FP-NDV (vFP-ND) by Taylor et al. [28].

As in studies 1 and 2, the heterologous prime-boost regimen induced higher HI titers in chicks with AI MDA than the inactivated vaccine alone. The vFP89 priming was efficient in birds with H5N9 MDA and override the negative effects of MDA on inH5N9 immunogenicity (Figure 4). In study 4, the protection of the prime-boost in H5N9 MDA was higher (7/9 protected; MDT 7.0) than that of inH5N9 in FP MDA (5/9 protected; MDT 7.3) or in H5N9 MDA (4/9 protected; MDT 5.0) confirming the positive effect of the vFP89 priming in both type of birds. Such priming effect of vectored vaccines has also been observed in a passive antibody transfer study mimicking MDA [12].

Among the different types of MDA tested (FP, H5N9 or H5(N8) + H5N9 + FP), the highest interference on the prime-boost immunogenicity (Figure 5) was observed at D28 in birds hatched from breeders vaccinated with the prime-boost regimen (H5(N8) + H5N9 + FP MDA) suggesting that, as for inactivated vaccine, interference is higher when the same vaccines and vaccination schemes are used in breeders and their progeny. Several mechanisms responsible for MDA interference have been proposed including (1) antigen removal by elimination of immune complexes, (2) neutralization of live vaccine virus, (3) epitope masking and (4) inhibiting of B-cells by cross-linking of B-cell receptor, antigen, maternal antibodies and the Fcγ receptor IIB. Kim et al. [33] found that it was the latter mechanism which was mainly responsible for interference in a measles vaccination model in cotton rats. Further studies are needed to better understand the mechanisms of MDA interference in chickens.

The data presented here and recent published work show that AI MDA can potentially interfere on both inactivated whole avian influenza virus and live fowl poxvirus vectored vaccines. Interestingly, the heterologous prime-boost gave the best immune response and the best level of protection. Similar fowlpox vector/inactivated vaccine prime-boost was shown to be highly immunogenic in ducks [34]. There are at least 3 explanations for the highest performance of such heterologous prime-boost [6,35]. Firstly, the two types of vaccine induce different type of immunity: the live fowlpox vector vaccine induces mainly cellular immunity and the inactivated vaccine induced mainly humoral immunity, the combination of two providing a more balanced Th1/Th2 and a broader immune response. Secondly, the fowlpox vector is expressing only HA, the primary protective antigen of influenza virus. After the boost with the whole virus inactivated vaccine, the higher secondary immune response will be directed against the protective HA antigen only and a lower primary immune response will be induced against the other antigens. The broader immunogenicity of a prime-boost regimen using a vector expressing the protective antigen and protein (or another vector) has been shown for different antigens [36] including recently for influenza in mice, ferrets and monkeys [37]. Thirdly, the HA inserted in the fowlpox vector (from an H5N8 isolate) is different from the HA present in the inactivated vaccine (from an H5N9 isolate). The secondary immune response after the inactivated vaccine boost will be directed against the epitopes common to the two HA antigens. These common epitopes (including epitopes from the HA stem) are thought to be more conserved than the strain specific epitopes resulting in a broader immune response [35]. Heterologous prime-boost regimens with vaccines containing different H5N1 antigens have been shown to provide broader immunity than homologous prime-boost ones with the same vaccine [38-40]. Such successive immunization with HA of different origins has also recently allowed to generate broadly reactive H3 monoclonal antibodies [41] and are used to induce broadly reactive antibodies recognizing the HA stalk [42]. Similar benefits of a prime-boost vaccination regimen in birds with MDA were recently shown in an HPAI H5N1 model with a herpesvirus of turkey vector priming [43].

Countries such as Indonesia and Egypt in which H5N1 HPAI infection became endemic have not eradicated the infection by vaccination. This failure has many explanations [44,45], including the suboptimal use of vaccines that seems to have led to the emergence of new antigenic variants against which the classical vaccines are not fully protective [3,16,46]. MDA interference on vaccine efficacy likely contributes also to the lack of control by vaccination [9,26]. Results presented here confirm the high MDA interference on inactivated vaccines and suggest that the use of a prime-boost strategy using a fowlpox vector to prime may be able to overcome at least partially MDA interference. Furthermore, the vaccines and vaccination protocol used in breeders and in their progeny need to be different to minimize MDA interference. Additional vaccination/challenge studies need to be done in field conditions to find the optimal vaccination schemes providing the best protection in breeders and in their progeny.

## Abbreviations

AI: Avian Influenza
D0: day 0
D14: day 14
D21: day 21
D28: day 28
D42: day 42
D84: day 84
dpc: days post-challenge
dpV1: days post first vaccination
dpV2: days post second vaccination
EID_50_: 50% Egg Infectious Dose
ELISA: enzyme-linked immunosorbent assay
FP: fowlpox
inH5N9: A/chicken/Italy/22A/98 H5N9 whole virus inactivated vaccine
HA: Hemagglutinin
HI: Hemagglutination inhibition
HPAI: Highly Pathogenic Avian influenza
LPAI: Low pathogenic avian influenza
MDA: Maternally-derived antibodies
MDT: Mean death time
NDV: Newcastle disease virus
PBS: Phosphate buffered saline
pch: post-challenge
SPF: Specific pathogen free
TCID50: 50% tissue culture infectious dose
vFP2211: Fowlpox-recombinant containing a synthetic A/chicken/Indonesia/7/2003 H5N1 HA gene modified at the cleavage site
vFP89: Fowlpox-recombinant containing the H5 of A/turkey/Ireland/83 (HP H5N8)
vFP-ND: Fowlpox-recombinant containing a NDV protective gene
